# Identification of unique biomarkers in colorectal cancer based on comprehensive analysis and machine learning

**DOI:** 10.3389/fonc.2025.1678750

**Published:** 2026-01-26

**Authors:** Liwei Wang, Aigang Ren, Xiaolong Cui, Yuan Shen, Qingxing Huang

**Affiliations:** Department of Colorectal and Anorectal Surgery, First Hospital of Shanxi Medical University, Taiyuan, China

**Keywords:** colorectal cancer, bioinformatics analysis, machine learning, biomarkers, ferroptosis

## Abstract

**Introduction:**

Colorectal cancer (CRC) is a common malignant tumor with high incidence and poor prognosis. Identifying effective biomarkers is crucial for its diagnosis and treatment.

**Methods:**

Gene expression data were obtained from TCGA-CRC and GSE39582 datasets. After preprocessing, differentially expressed genes (DEGs) were screened using the limma package. Hub genes were identified via WGCNA, miRNA-hub/TF-hub gene network construction, and LASSO, SVM-RFE, and random forest algorithms. Subtype analysis, survival analysis, external validation, qRT-PCR, Western blot, and ferroptosis-related assays were performed.

**Results:**

Fourteen ferroptosis-mitochondria-RBP-related genes (IMRBPs) were identified, including seven hub RBP genes (APEX1, BRCA1, DNMT1, EZH2, PTTG1, SND1, UHRF1). APEX1 was downregulated in CRC, while the other six were upregulated. The diagnostic model based on these seven genes showed high AUC values (0.818-0.924) in multiple datasets. These hub genes were associated with ferroptosis suppression by regulating GSH/GSSGand Fe²⁺ levels.

**Discussion:**

The seven hub RBP genes are potential biomarkers for CRC, providing new insights and therapeutic targets. However, functional validation and larger sample sizes are needed for clinical application.

## Introduction

Colorectal cancer (CRC), a common malignant tumor, has a high incidence rate worldwide and seriously threatens human health (1, 2). Its onset is insidious, and in the early stage, there are often no obvious symptoms (3). Most patients are already in the middle and late stages when diagnosed (4), which brings great challenges to treatment and also leads to a relatively poor prognosis for colorectal cancer patients. Therefore, in-depth exploration of the pathogenesis of colorectal cancer and the search for effective early diagnostic markers and therapeutic targets have become the current research hotspots and difficulties.

Similar attempts have been made to identify diagnostic biomarkers for CRC using different models and approaches. For instance, a recent study employed a distinct bioinformatics pipeline to identify key genes and construct diagnostic models, demonstrating the feasibility and value of computational approaches in CRC biomarker discovery (5). Recent studies have highlighted the potential role of GLP-1 receptor agonists in colorectal cancer. GLP-1 receptor agonists, commonly used in type 2 diabetes management, have been associated with a reduced risk of CRC development and progression, suggesting their potential repurposing as adjunctive therapeutic agents (6). Additionally, microRNAs play crucial roles in CRC pathogenesis. For instance, the polymorphism miR-27 rs895819 has been implicated in altering individual susceptibility to CRC, while miR-423 expression levels are associated with tumor progression and patient prognosis, serving as potential diagnostic and prognostic biomarkers (7). Furthermore, the ribosomal protein L22-like 1 (RPL22L1) has been identified as a novel player in CRC, where its dysregulation influences tumor growth and metastasis, highlighting its potential as a therapeutic target (8). The integration of network analysis approaches, as exemplified by robust methodologies in systems biology (9), provides a powerful framework for elucidating complex molecular interactions in cancer, offering insights into hub genes and key regulatory networks. Non-coding RNAs, such as the long intergenic non-coding RNA linc01615, have been shown to promote CRC survival and metastasis by sponging miR-491-5p, thereby modulating downstream oncogenic pathways (10). These findings collectively underscore the complexity of CRC pathogenesis and the importance of multifaceted biomarker discovery.

RNA-binding proteins (RBPs) (11, 12) play a key regulatory role in the RNA metabolism process within cells (13, 14), and their abnormal functions are closely related to the occurrence and development of many diseases (15–18). Ferroptosis, as a novel form of programmed cell death, has a unique mechanism and is different from traditional apoptosis, necrosis, and autophagy and may play an important role in the process of tumorigenesis and development (19–24). The mitochondria, as the energy factory of cells, are not only responsible for energy production but also participate in many biological processes such as apoptosis, signal transduction, and metabolism, whose dysfunction is also closely related to tumorigenesis (25–28).

In recent years, the rapid development of bioinformatics analysis and machine learning technology has provided powerful means for mining potential biomarkers from vast amounts of gene data (29, 30). Based on this, this study integrated the gene expression profile data from the TCGA database and the GSE39582 dataset and applied a variety of advanced bioinformatics methods, including strict data preprocessing, screening of differentially expressed genes, weighted gene co-expression network analysis (WGCNA), construction of miRNA-hub gene networks and TF-hub gene networks, and screening of hub genes using LASSO and SVM-RFE algorithms, aiming to systematically study the expression characteristics, interrelationships, and potential clinical significance of RBP, mitochondrial, and ferroptosis-related genes in colorectal cancer, with the expectation of identifying unique biomarkers with diagnostic and therapeutic value, opening up new directions for colorectal cancer research, and providing a theoretical basis for improving the clinical outcome of colorectal cancer patients.

## Materials and methods

### Data preparation and preprocessing

The gene expression profile data of colorectal cancer were downloaded from the TCGA database (https://www.cancer.gov/ccg/research/genome-sequencing/tcga) and the GSE39582 dataset (http://www.ncbi.nlm.nih.gov/geo) as the training set and validation set. The TCGA training set contains 283 tumor samples and 42 normal samples, and the GSE39582 dataset contains 566 tumor samples and 19 normal samples (**Table 1**). The GEO data were processed by deleting probes for the empty carrier, removing probes corresponding to multiple genes, and taking the median value when multiple probes correspond to the same gene. The ferroptosis dataset was downloaded from http://zhounan.org/ferrdb/current/, containing a total of 564 genes. A total of 1,136 human mitochondrial genes were obtained from the MitoCarta3.0 database, and 5,346 human RBP genes were obtained from the RBPDB website (http://rbpdb.ccbr.utoronto.ca/advanced_search.php), Gerstberger, SONAR, the Gene Ontology project, Poly(A) binding protein, CARIC, and XRNAX online software (31).

**Table 1:** GEO/TCGA data tumor and normal sample statistics.

Data	Tumor	Normal
TCGA-CRC	283	42
GSE39582	566	19

### Screening of differentially expressed genes

The limma package in the R software was used to obtain differentially expressed genes (DEGs) from the training set TCGA-CRC based on the threshold *P <*0.05. The DEGs were then visualized using R packages (dplyr, ggplot2, ggrepel) in volcano plots.

### WGCNA analysis

WGCNA was performed to explore the correlation and potential regulatory relationships among RBP genes, mitochondrial genes, and ferroptosis genes.

In our study, WGCNA was constructed by the R package “WGCNA” on the TCGA-CRC transcriptional expression profile, and the distribution map of the soft threshold and average connectivity was drawn. Specifically, the enrichment scores of samples for ferroptosis genes, mitochondrial genes, and RBP gene sets were calculated through ssGSEA. In order to ensure that the network is a scale-free network, the threshold for the scale-free fitting index was set to 0.8. The average-linkage hierarchical clustering method was applied to cluster genes, following the standard of the mixed dynamic shear tree, and the minimum number of genes in each gene network module was set to 30. Based on the sample ssGSEA enrichment score and the top 25% of the absolute median difference of the TCGA-CRC patient expression profile, a weighted gene co-expression network was constructed to identify whether the strongly correlated co-expression modules between the RBP genes and the ferroptosis mitochondrial genes in TCGA-CRC were consistent and to preliminarily infer whether there was a regulatory relationship among them.

### Construction of the miRNA-hub gene network and the TF-hub gene network

The RBP genes related to chronic sinusitis were obtained by the intersection of RBP genes and DEGs. The TRRUST (version 2) (https://www.grnpedia.org/trrust/) online human transcription factor database was used to retrieve the transcription factors related to differentially expressed RBP genes, ferroptosis genes, and mitochondrial-related genes.

Firstly, we obtained the common transcription factor (TF) regulatory network of the key genes of the RBP module, ferroptosis genes, and mitochondrial-related genes. Then, the differentially expressed RBP genes, ferroptosis genes, and mitochondrial-related genes in this regulatory network were used to perform machine learning and to obtain the key genes as the key regulatory network.

### Screening hub genes by LASSO, SVM-RFE, and random forest algorithms

The R packages “glmnet,” “caret,” and “randomForest” were used to perform least absolute shrinkage and selection operator (LASSO), support vector machine-recursive feature elimination (SVM-RFE), and random forest (RF) algorithm analysis on the differentially expressed RBP genes, ferroptosis genes, and mitochondrial-related genes in the above network using the training set. For LASSO, 10-fold cross-validation was performed to determine the optimal lambda value. For SVM-RFE, a linear kernel was used with five-fold cross-validation repeated three times. For random forest, 1,000 trees were grown with the mtry set to the square root of the number of predictors. A fixed random seed was set for reproducibility. The final differentially expressed RBP, ferroptosis, and mitochondrial hub genes were obtained by taking an intersection of the genes screened by the LASSO, SVM-RFE, and RF methods.

### Nested cross-validation for machine learning model optimization

To avoid overfitting and ensure the robustness of the diagnostic model based on the seven hub RNA-binding protein (RBP) genes (APEX1, BRCA1, DNMT1, EZH2, PTTG1, SND1, UHRF1), nested cross-validation (NCV) was performed on the TCGA-CRC training set (283 tumor samples, 42 normal samples). The NCV framework consisted of an outer loop and an inner loop, both implemented using the caret package in R (version 4.3.0).

### Survival analysis (OS/DFS)

Clinical information and gene expression data from the TCGA-CRC dataset were used, including overall survival (OS) and disease-free survival (DFS) of 283 CRC patients. Samples with unknown survival status, missing follow-up time, or incomplete clinical information were excluded, and a total of 268 valid samples were retained for survival analysis.

### External dataset validation (GSE14333, GSE17536)

Gene expression data of GSE14333 (130 samples: 110 CRC tissues, 20 normal tissues) and GSE17536 (168 samples: 140 CRC tissues, 28 normal tissues) were downloaded from the GEO database. The same preprocessing pipeline as that for the TCGA-CRC dataset was applied: deletion of empty vector probes, merging of expression values of multiple probes corresponding to the same gene (taking the median), and standardization (*z*-score normalization). Based on the diagnostic model of the seven hub RBP genes established using the TCGA-CRC dataset, the risk score of each sample in the GSE14333 and GSE17536 datasets was calculated. The pROC package in R software was used to generate ROC curves, and the AUC value, sensitivity, specificity, and 95% confidence interval were calculated to verify the diagnostic efficacy of the model in external cohorts.

### PPI network construction

The intersecting genes (TF genes, RBP genes, ferroptosis genes, and mitochondria genes) were applied to establish protein–protein interaction (PPI) networks by the STRING database (https://cn.string-db.org/, version: 12.0), which was visualized by Cytoscape. A *P*-value <0.05 was considered statistically significant.

### Hierarchical clustering analysis

Based on the RBP hub gene, the R package ConsensusClusterPlus (32) was used for unsupervised clustering. First, the samples were divided into two categories. Then, K-means and Euclidean distance were selected, and the class with the most moderate decline was selected as the best cluster number according to the CDF decline curve.

The ssGSEA method of the R package GSVA (33) was applied to estimate the score of the sample in each hallmark pathway, and then the R package ComplexHeatmap was used to draw the enrichment score distribution of different hallmark pathways between subtypes.

### GSEA analysis

We performed a single-gene GSEA analysis (R GSVA package) to investigate the possible roles of hub genes.

### Functional enrichment analysis

R package (clusterProfiler, enrichplot) (34) was used to perform Gene Ontology (GO) and the Kyoto Encyclopedia of Genes and Genomes (KEGG) pathway analysis of the hub RBP genes.

### Assessment of immune cell infiltration

The marked immune infiltrating cell type was obtained from the study of Charoentong (35), which includes human immune cell subtypes, such as activated CD8 T cells, activated dendritic cells, macrophages, natural killer T cells, and regulatory T cells. Then, the R package (IOBR and GSVA) (36) was explored to calculate the scores of immune cells in the sample microenvironment.

### Signature-related molecular characteristics and drug responses

The R packet oncoPredict (37) was used to predict the Spearman correlation between the log2(IC_50_) of each drug in various cell lines in the GDSC2 database, based on the expression data and drug response information of cell lines in the GDSC2 database, combined with the training set expression profile.

### Stemness score analysis of hub genes

The mRNA stemness index (mRNAsi) was calculated using the OCLR algorithm constructed by Malta et al. (38). The minimum was subtracted, and the result was divided by the maximum, mapping the stemness index to a range of 0 to 1. The distribution of OCLR scores was shown from low to high, and the Kruskal–Wallis test was used to test the significance of differences.

### Patients’ samples

Tissue samples from 50 colorectal cancer patients and 50 non-tumor tissue samples were collected for the study. All patients signed informed consent forms, and the collection, processing, and analysis of the samples were conducted under the guidance of the Ethics Committee of the First Hospital of Shanxi Medical University (Ethics Approval KYLL-2024-346).

### Quantitative real-time PCR and Western blot analysis

The total tissue RNA was extracted using TRIzol reagent (Invitrogen, Carlsbad, CA, USA), followed by reverse transcription of the total RNA into complementary DNA (cDNA) using the cDNA reverse transcription kit (Yisheng Biotechnology, 11119ES60, Shanghai, China). Quantitative real-time polymerase chain reaction (qRT-PCR) was conducted using the qPCR SYBR Green kit (Yisheng Biotechnology, 11203ES03, Shanghai, China). The primer sequences for the TF genes, RBP genes, ferroptosis genes, and mitochondria genes are shown in **Table 2**. The GAPDH gene was utilized as an internal reference gene. Each biological sample was subjected to three technical replicates.

**Table 2:** The sequences of the qPCR primers used in the research.

Gene	Primer	Sequences 5′–3′
GAPDH	Forward primer	5′-CTCACCGGATGCACCAATGTT-3′
	Reverse primer	5′-CGCGTTGCTCACAATGTTCAT-3′
UHRF 1	Forward primer	5′-ATGGGTTTTGACTGATTCCCAG-3′
	Reverse primer	5′-GCAGATGCGACTACTGTAGAGC-3′
SND1	Forward primer	5′-CTTCTGCCCCGACATCTTTCT-3′
	Reverse primer	5′-GCCGACAGGTCACTACAAGG-3′
PTTG1	Forward primer	5′-ACCCGTGTGGTTGCTAAGG-3′
	Reverse primer	5′-ACGTGGTGTTGAAACTTGAGAT-3′
BRCA1	Forward primer	5′-AGGAGCAAAACGACTAGCTGC-3′
	Reverse primer	5′-TCTGGGGTCTTCATGTCTGAT-3′
EZH2	Forward primer	5′-GGACCACAGTGTTACCAGCAT-3′
	Reverse primer	5′-GTGGGGTCTTTATCCGCTCAG-3′
DNMT 1	Forward primer	5′-AGAACGGTGCTCATGCTTACA-3′
	Reverse primer	5′-CTCTACGGGCTTCACTTCTTG-3′
APEX1	Forward primer	5′-GTTTCTTACGGCATAGGCGAT-3′
	Reverse primer	5′-CACAAACGAGTCAAATTCAGCC-3′

The total tissue protein was isolated using T-PER Tissue Protein Extraction Reagent (Thermo Fisher, 78510). Additionally, the procedure refers to a previous protocol (39). The dilution of primary antibodies is shown in **Table 3**.

**Table 3:** The antibodies of WB used in the research.

Antibodies	Dilution	Manufacturer	Cat. no.
UHRF1 (D6G8E) rabbit mAb	1:1,000	CST	12387
Anti-SND1	1 μg/mL	Abcam	Ab65078
Anti-PTTG1IP	1:1,000	Abcam	Ab128040
BRCA1 (E5S9G) rabbit mAb	1:1,000	CST	50799
Recombinant anti-KMT6/EZH2 [EPR20108] - ChIP grade	1:1,000	Abcam	Ab191250
Recombinant anti-Dnmt1	1:1,000	Abcam	ab188453
Recombinant Anti-APE1 [EPR18378-45] - ChIP grade	1:1,000	Abcam	ab189474
Anti-GAPDH	1:5,000	Abcam	ab8245
HRP * polyclonal goat Anti-mouse IgG	1:5,000	Sevier	GB23301
HRP * polyclonal goat Anti-rabbit IgG	1:3,000	Sevier	GB23303

* P < 0.05.

### Glutathione/glutathione disulfide assay

The total glutathione (T-GSH)/oxidized glutathione (GSSG) Assay Kit (microplate method) (A061-1-2, Jiancheng Bioengineering Institute, China) was used to perform the glutathione (GSH) and GSSG assay. According to the instructions, we prepared the required reagents and the GSH/GSSG standard sample. After mixing the tissues with homogenization medium and centrifugation, the supernatant was mixed with the above reagents and the standard sample. The absorbance increments of the resulting mixture at 30 s and 10 min 30 s were then measured using a microplate reader at 405 nm. Then, we calculated the levels of GSSG and GSH according to the formula provided by the manufacturer.

### Ferroptosis (Fe^2+^) level assay

The protein samples were firstly extracted and isolated from the tumor tissues and qualified by a Pierce BCA protein detection kit (PC0020, Solarbio, Beijing, China). The detection of tissue ferroptosis was conducted by a tissue iron (Fe) assay kit purchased from Jiancheng Bioengineering Institute (A039-2-1, Nanjing, China) according to the manufacturer’s recommended protocol.

### Malondialdehyde assay

The intracellular malondialdehyde (MDA) concentrations were determined using the lipid oxidation (MDA) assay kit (S0131S, Biyuntian, China) according to the manufacturer’s recommended protocol.

### Statistical analysis

All statistics were performed using the R software (version 4.1.2; https://www.R-project.org) and GraphPad Prism 9. The Wilcoxon test was used to screen for statistically differentially expressed genes and infiltrating immune cells. The heatmap and volcano plot of differentially expressed genes were drawn using pheatmap and ggplot2, respectively. The Venn diagram was drawn using the ggVenn package. Statistical analyses of qRT-PCR, WB, free iron content, GSH, GSSG, and MDA were presented as the mean ± standard deviation for at least five individual experiments, and the statistical significance of differences was determined with the unpaired, two-tailed Student’s *t*-test. A *P*-value <0.05 is considered statistically significant for all analyses (****P < 0.0001; ***P < 0.001; **P < 0.01; *P < 0.05; ns indicates not significant).

## Results

### Screening of differentially expressed genes

**Figure 1:**
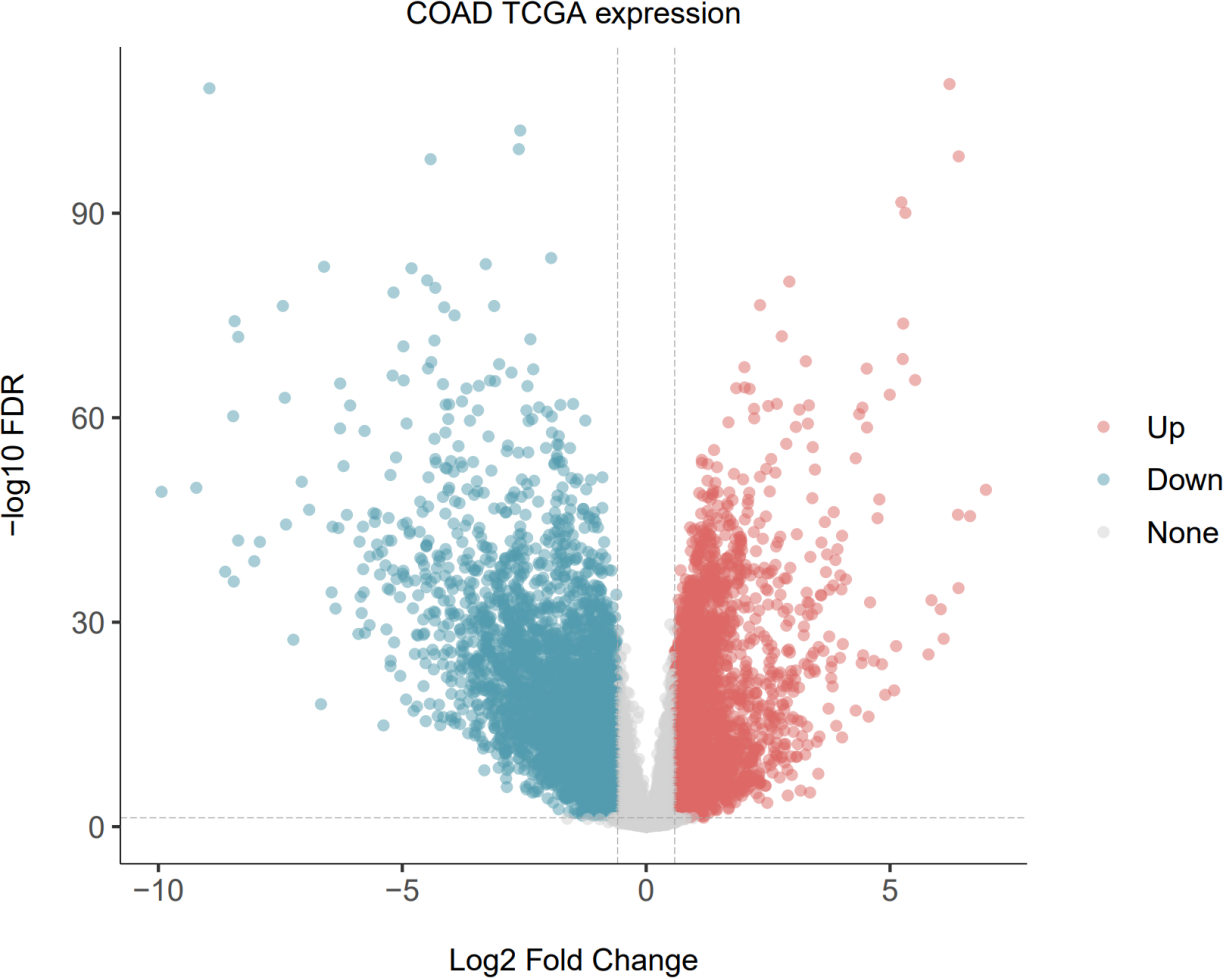
Volcano plot of differentially expressed genes in the TCGA-CRC dataset.

After preprocessing raw data, genes with *P*-value <0.05 and |log2FC| >1 were considered DEGs. We obtained 4,463 DEGs, which included 1,454 upregulated genes and 3,009 downregulated genes. A volcano plot for the differential analysis is shown in **Figure 1**.

### WGCNA network construction and CRC-related module identification

**Figure 2:**
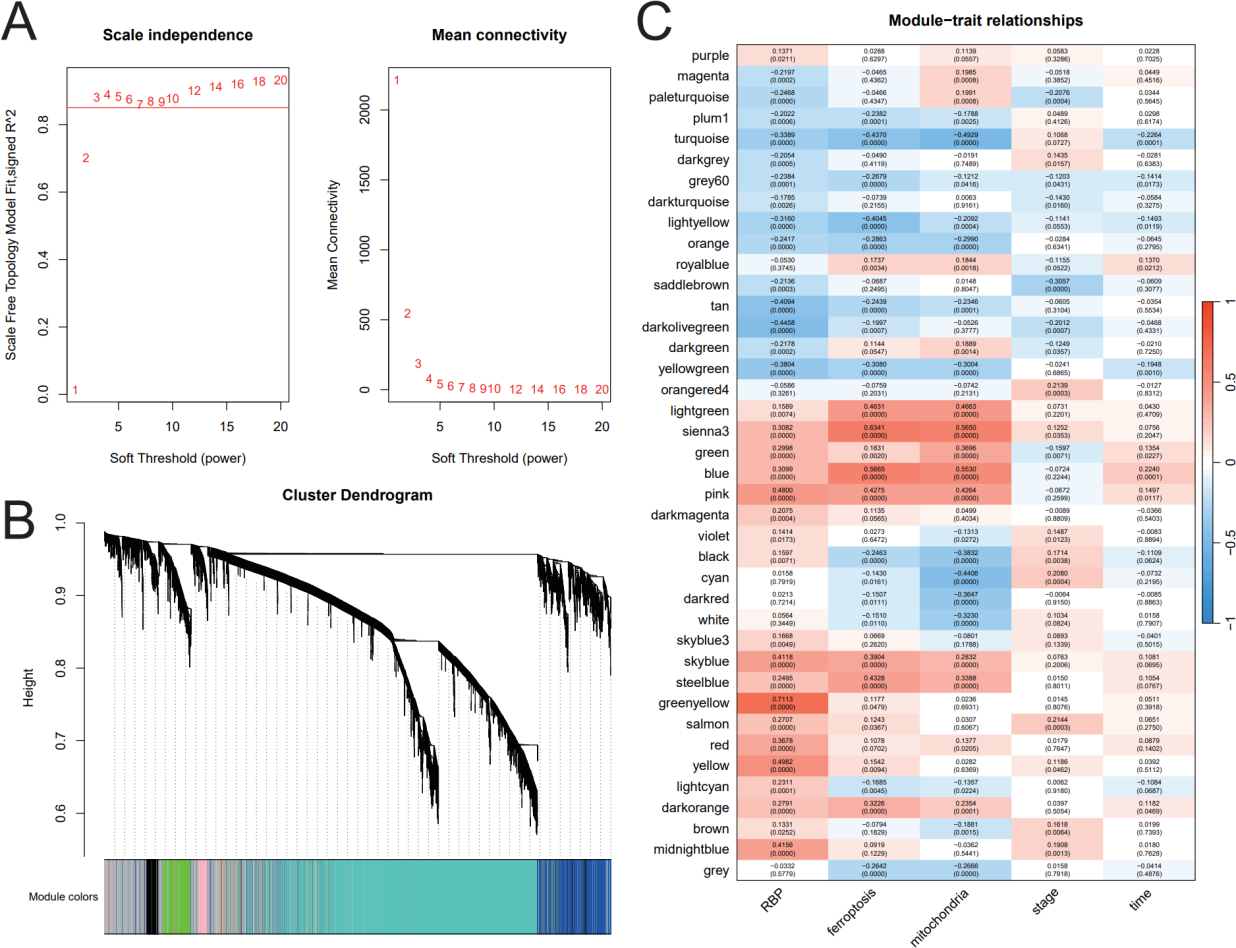
Weighted gene co-expression network analysis (WGCNA) network construction and CRC-related module identification. **(A)** Analysis of free-scale network topology for different soft-thresholding powers. **(B)** Hierarchical clustering plot showing the relationships of genes within modules. **(C)** Heatmap showing the relationship between ferroptosis phenotype and each module: red represents a positive correlation, and blue represents a negative correlation.

To determine whether potential gene modules are associated with CRC, we performed a WGCNA analysis of all candidate genes in the ferroptosis, mitochondrial, and RBP-related dataset. To build a scale-free network, we choose a soft threshold = 3, with a scale-free topology fitting index *R*^2^ >0.8 (**Figure 2A**). Furthermore, we used a one-step approach for module identification and merging (**Figure 2B**). The heatmap of the module–sample relationships showed that the co-expression module with the strongest correlation with the ferroptosis genes and mitochondrial genes is the same module. The correlation between the ferroptosis and mitochondrial-related genes and the module is very synchronous, but the correlation between the RBP and the module is somewhat different from them, and the relationship between them is further explored (**Figure 2C**).

### Screening hub genes by machine learning and PPI networks

**Figure 3:**
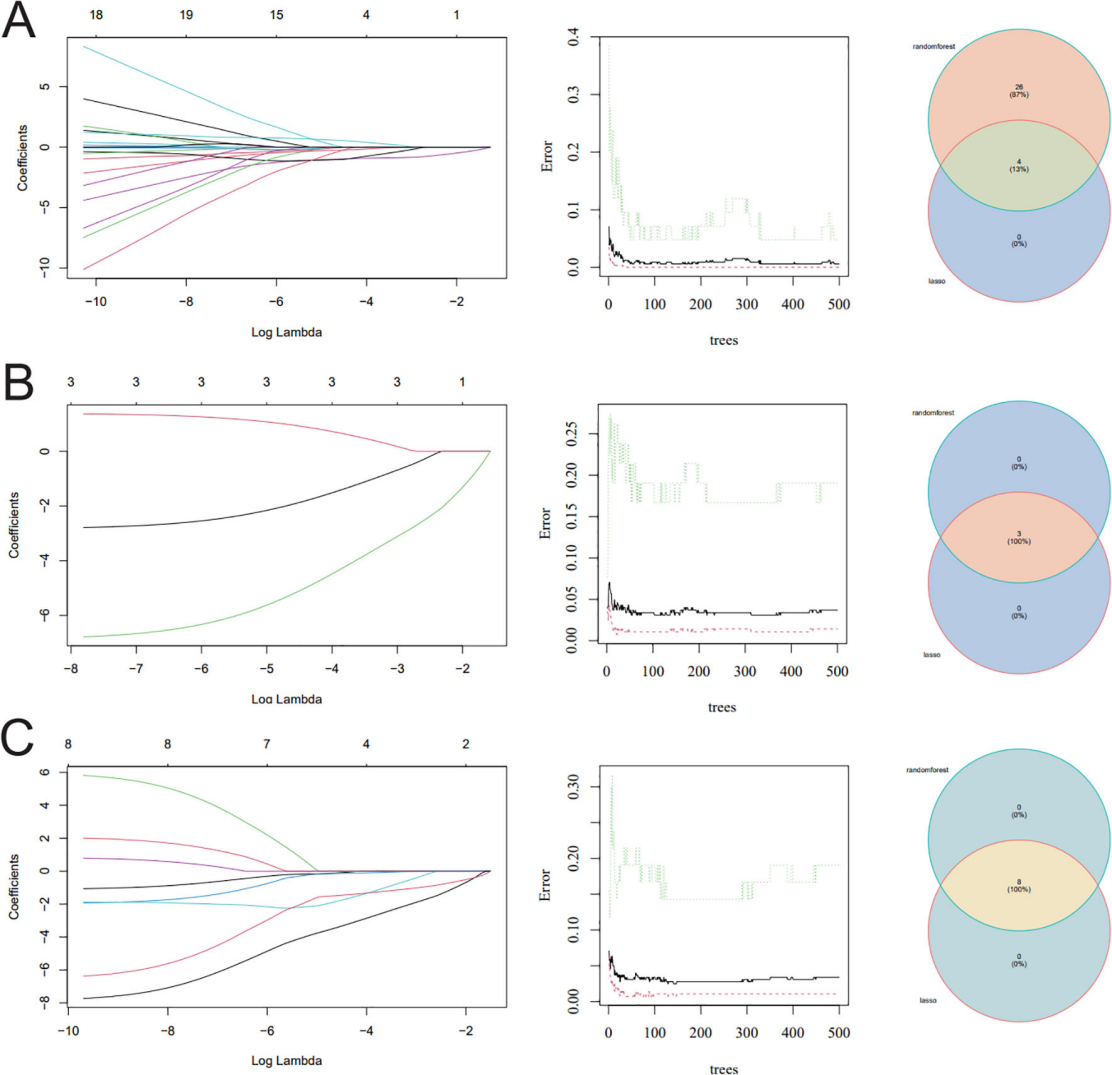
Screening hub genes by machine learning. (**A–C**) LASSO regression algorithm, RF algorithm, and Venn diagrams for the three algorithms. LASSO, least absolute shrinkage and selection operator; RF, random forest.

**Figure 4:**
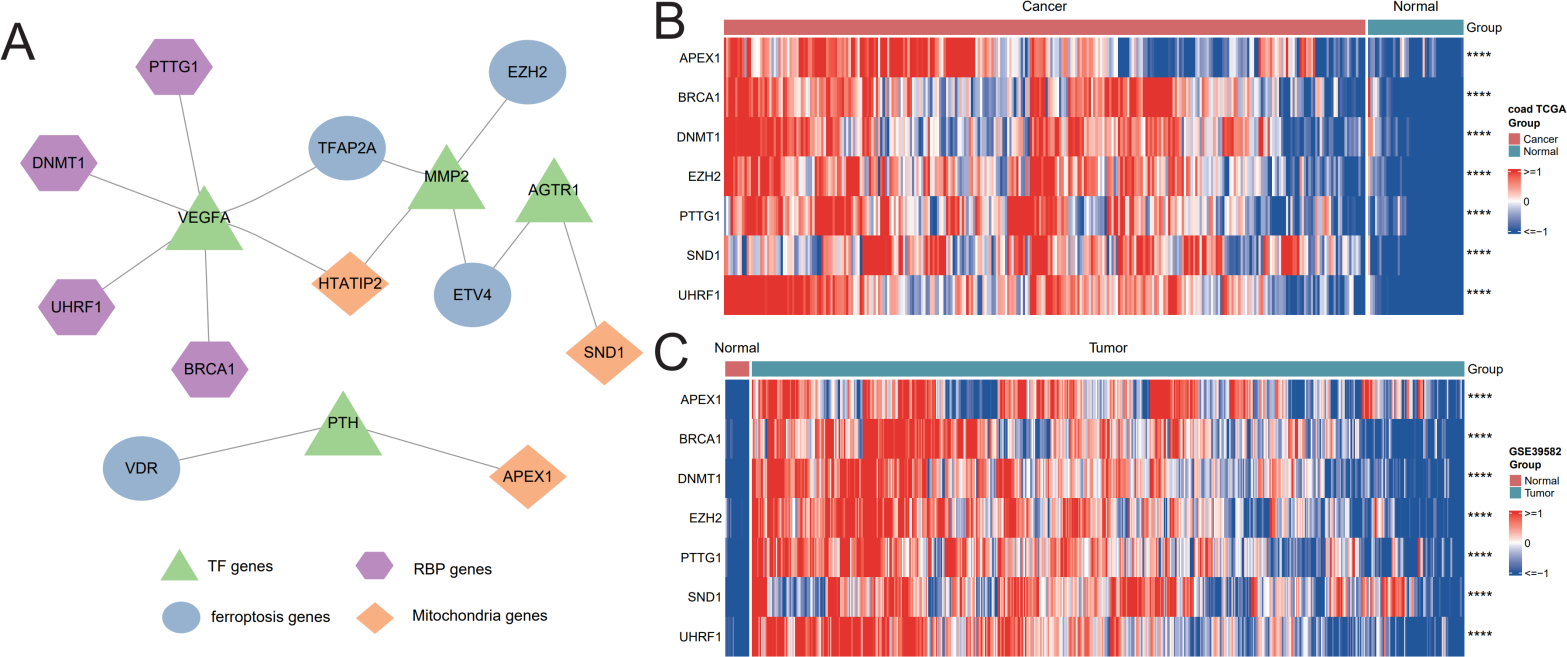
Screening hub genes by the PPI network and heatmap. **(A)** Regulatory network of the TF genes, RBP genes, ferroptosis genes, and mitochondria genes. **(B)** Heatmap of the seven hub RBP genes in the TCGA-CRC training set and GSE39582. **** P < 0.0001.

Firstly, four overlapped ferroptosis genes (VDR, TFAP2A, ETV4, and EZH2) were extracted using the LASSO regression algorithm and RF algorithm (**Figure 3A**). Secondly, three overlapped mitochondrial genes (APEX1, HTATIP2, and SND1) were identified using the LASSO regression algorithm and RF algorithm (**Figure 3B**). Then, eight RBP-related genes (APEX1, BRCA1, DNMT1, EZH2, PREB, PTTG1, SND1, and UHRF1) were selected by the LASSO regression algorithm and RF algorithm (**Figure 3C**). The obtained four ferroptosis genes, three mitochondrial genes, and eight RBP genes were used to make the public TF network map (**Figure 4A**), leaving four ferroptosis genes, three mitochondrial genes, and seven RBP genes (**Table 4**).

**Table 4:** The TF network map results of the ferroptosis, mitochondrial, and RBP genes.

RBP_gene	TF	Ferroptosis_gene	Mitochondrial_gene
APEX1	PTH	VDR	APEX1
BRCA1	VEGFA	TFAP2A	HTATIP2
BRCA1	VEGFA	TFAP2A	HTATIP2
DNMT1	VEGFA	TFAP2A	HTATIP2
DNMT1	VEGFA	TFAP2A	HTATIP2
EZH2	MMP2	ETV4	HTATIP2
EZH2	MMP2	EZH2	HTATIP2
EZH2	MMP2	TFAP2A	HTATIP2
PTTG1	VEGFA	TFAP2A	HTATIP2
PTTG1	VEGFA	TFAP2A	HTATIP2
SND1	AGTR1	ETV4	SND1
UHRF1	VEGFA	TFAP2A	HTATIP2
UHRF1	VEGFA	TFAP2A	HTATIP2

As a result, we identified 14 iron, mitochondrial, and RBP-related genes (IMRBPs). The seven hub RBP genes were APEX1, BRCA1, DNMT1, EZH2, PTTG1, SND1, and UHRF1. These seven key gene expression profiles in the TCGA-CRC training set and GSE39582 dataset were also visualized by a heatmap (**Figures 4B, C**). Finally, these seven hub RBP genes were selected as key genes for the following study.

### Subtype identification of hub RBP genes, molecular characteristics of hub genes, and drug response

**Figure 5:**
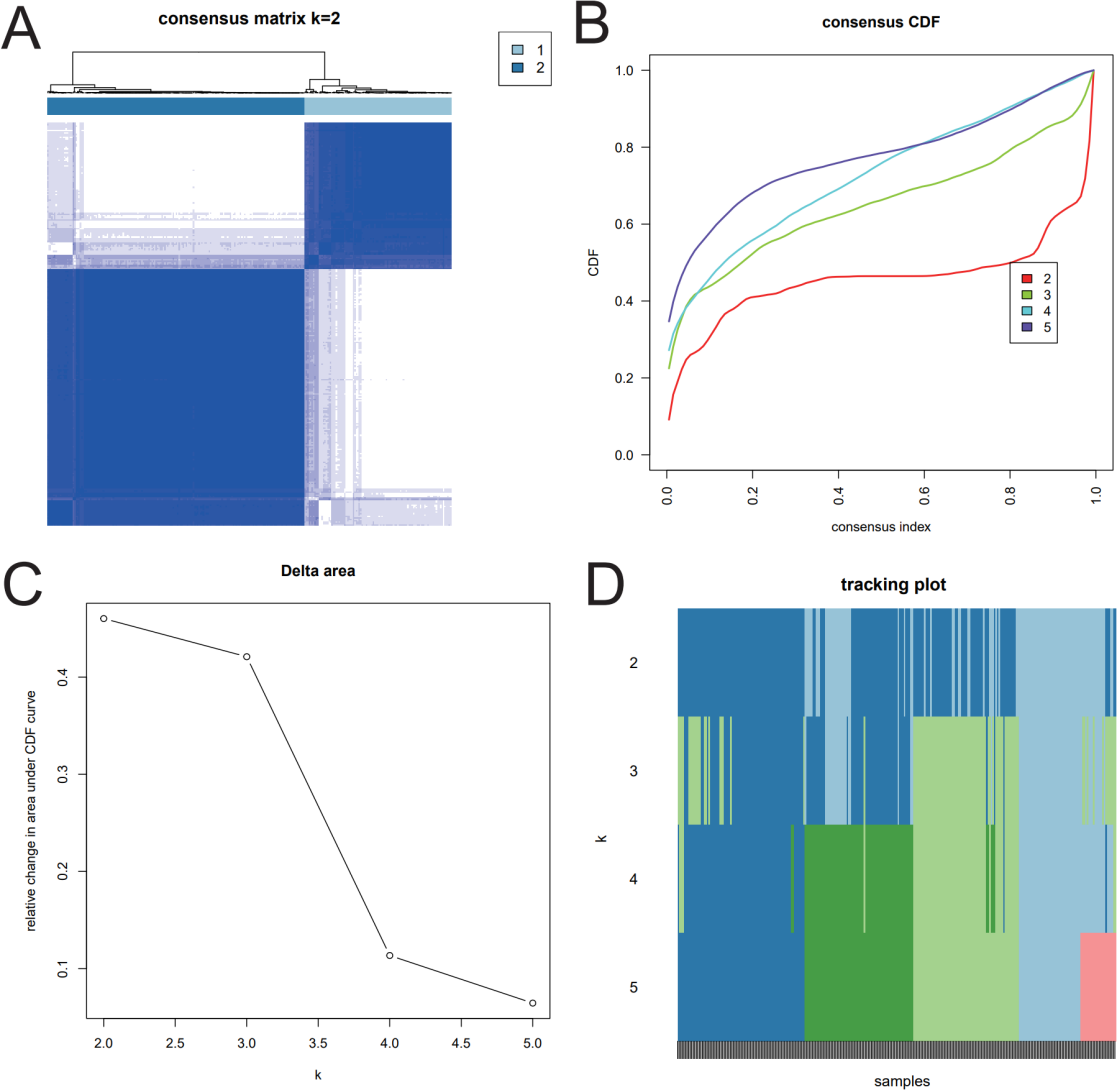
Heatmap of the seven hub RBP genes. **(A–D)** Sample clustering of the TCGA-CRC training set was performed in the seven hub RBP genes.

Based on the hub RBP genes, unsupervised clustering was used for the training set TCGA samples, and the optimal cluster number 2 was used as the subtype classification. The clustering results are shown in **Figures 5A–D**, and the results were clustered into two categories.

### Molecular characteristics and drug response of the hub IMRBP gene subtypes

**Figure 6:**
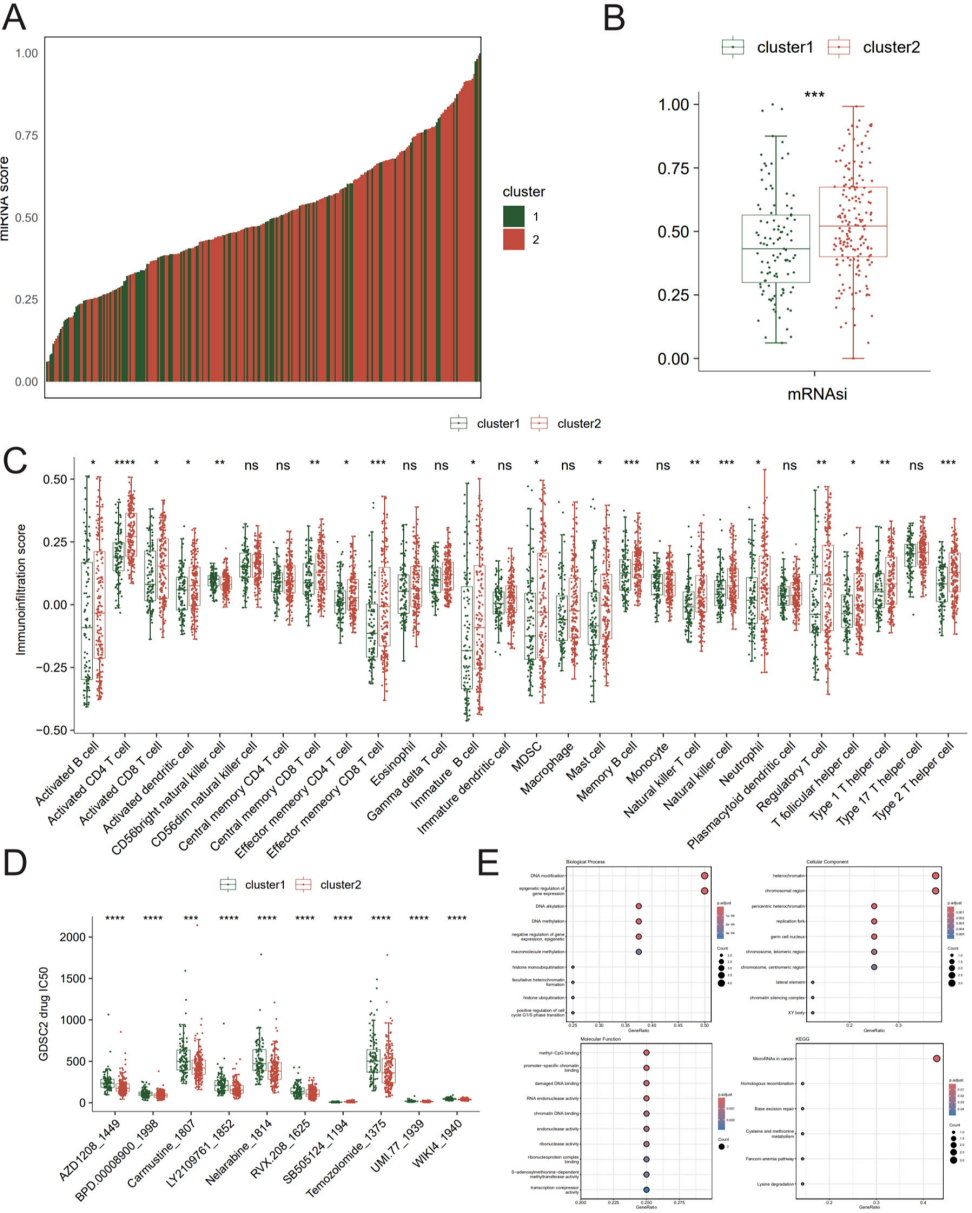
Molecular characteristics and drug response of the hub RBP gene subtypes. **(A, B)** Analysis of the stemness index of TCGA-CRC subtypes. **(C)** Box plot of differences in immune infiltration scores between subtypes. **(D)** Differences in the GCSD2 drug analysis between subtypes. **(E)** Results of the GO and KEGG enrichment analysis of hub RBP genes. * P < 0.05; ** P < 0.01; *** P < 0.001; **** P < 0.0001.

The results of the analysis of the stemness index between the two subtypes are shown in **Figures 6A, B**. Furthermore, the results of immune infiltration are shown in **Figure 6C**, including activated B cells, activated CD4 T cells, activated CD8 T cells, and CD56 bright natural killer cells, which were demonstrated to be comparable between the two clusters using the CIBERSORT algorithm. Ten potential therapeutic agents were screened in the GDSC2 database with a cutoff *P*-value of <0.05 (**Figure 6D**).

The results of the GO and KEGG analysis of the seven hub RBP genes were plotted as a bar chart (**Figure 6E**). In the GO analysis of these seven hub genes, 10 aspects were enriched in BP, including “DNA modification, epigenetic regulation of gene expression, DNA alkylation”; in CC, the central enrichment was in 10 areas such as “heterochromatin”; in MF, it was mainly enriched in “methyl-CpG binding,” “promoter-specific chromatin binding,” and “damaged DNA binding”; and in KEGG, the central enrichment was in six areas, including “microRNAs in cancer.”

**Figure 7:**
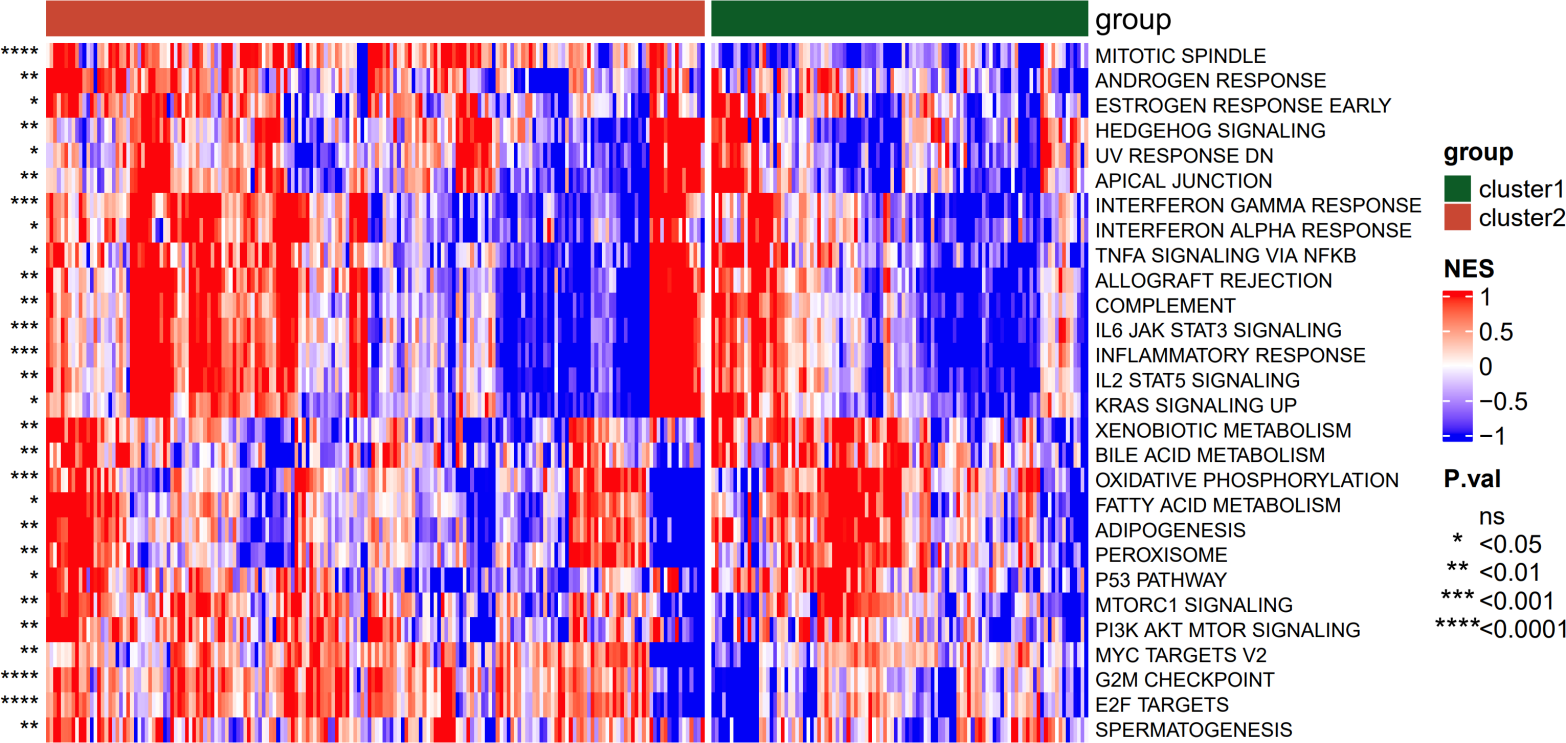
Results of the hallmark enrichment analysis between subtypes. * P < 0.05; ** P < 0.01; *** P < 0.001; **** P < 0.0001.

The results of the significant differences in enrichment of the hallmark channel are shown in **Figure 7**. In the two clusters, they were mainly enriched in the mitotic spindle, androgen response, and estrogen response early.

### Survival analysis results of the 14 IMRBP genes

**Figure 8:**
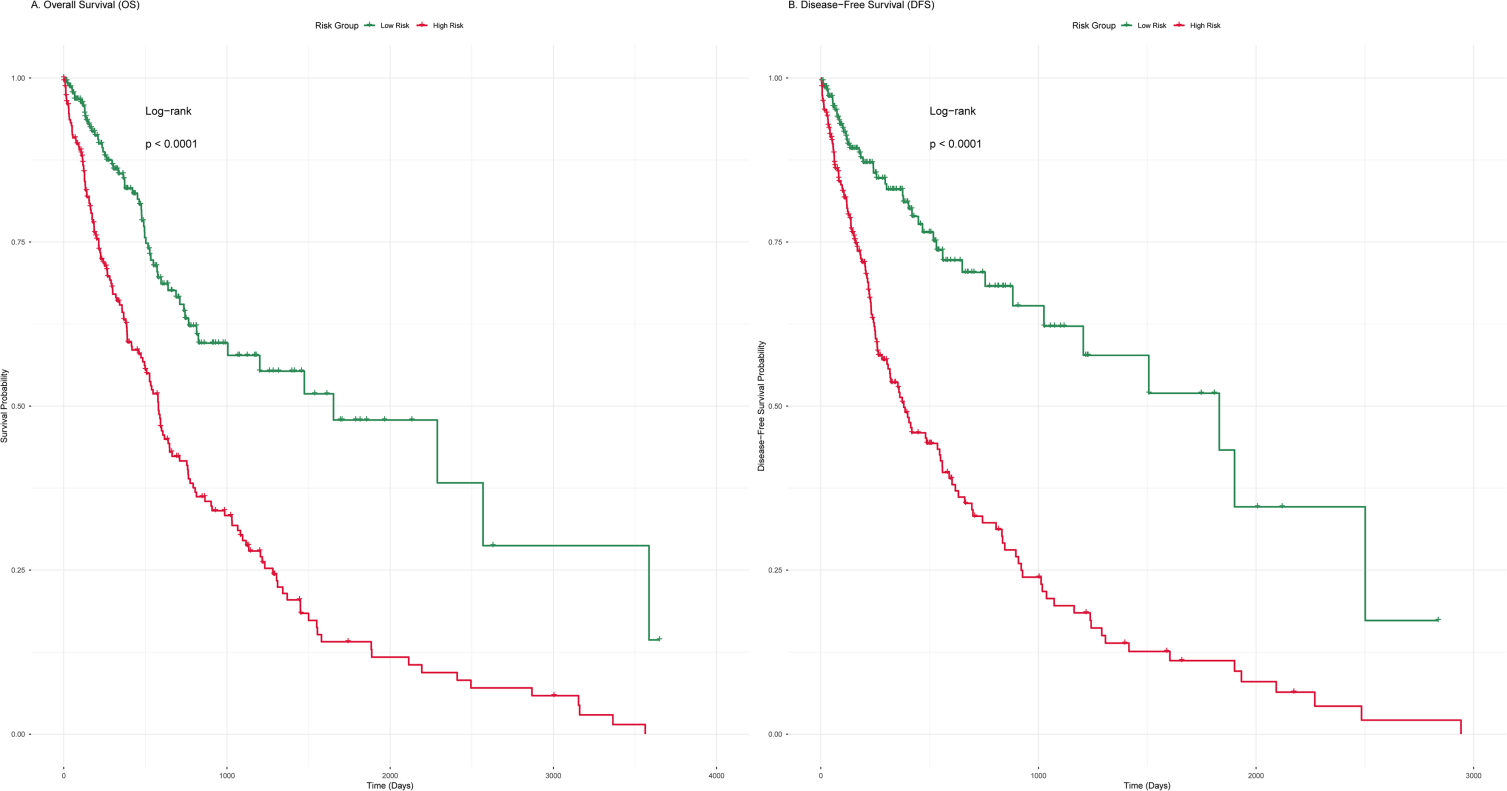
The OS and DFS of the expression of IMRBP genes between the high-risk group (red curve) and the low-risk group (blue curve), log-rank *P <*0.0001. **(A)** OS. **(B)** DFS.

Kaplan–Meier survival analysis showed that the OS of patients in the high-risk group was significantly shorter than that in the low-risk group (log-rank *P* < 0.0001), indicating that high expression of the 14 IMRBP genes was closely associated with poor OS in CRC patients (**Figure 8A**). The results of DFS analysis showed that the DFS of patients in the high-risk group was significantly lower than that in the low-risk group (log-rank *P* < 0.0001), suggesting that the IMRBP gene expression signature could effectively predict the recurrence risk of CRC patients (**Figure 8B**).

### Consistency analysis

**Figure 9:**
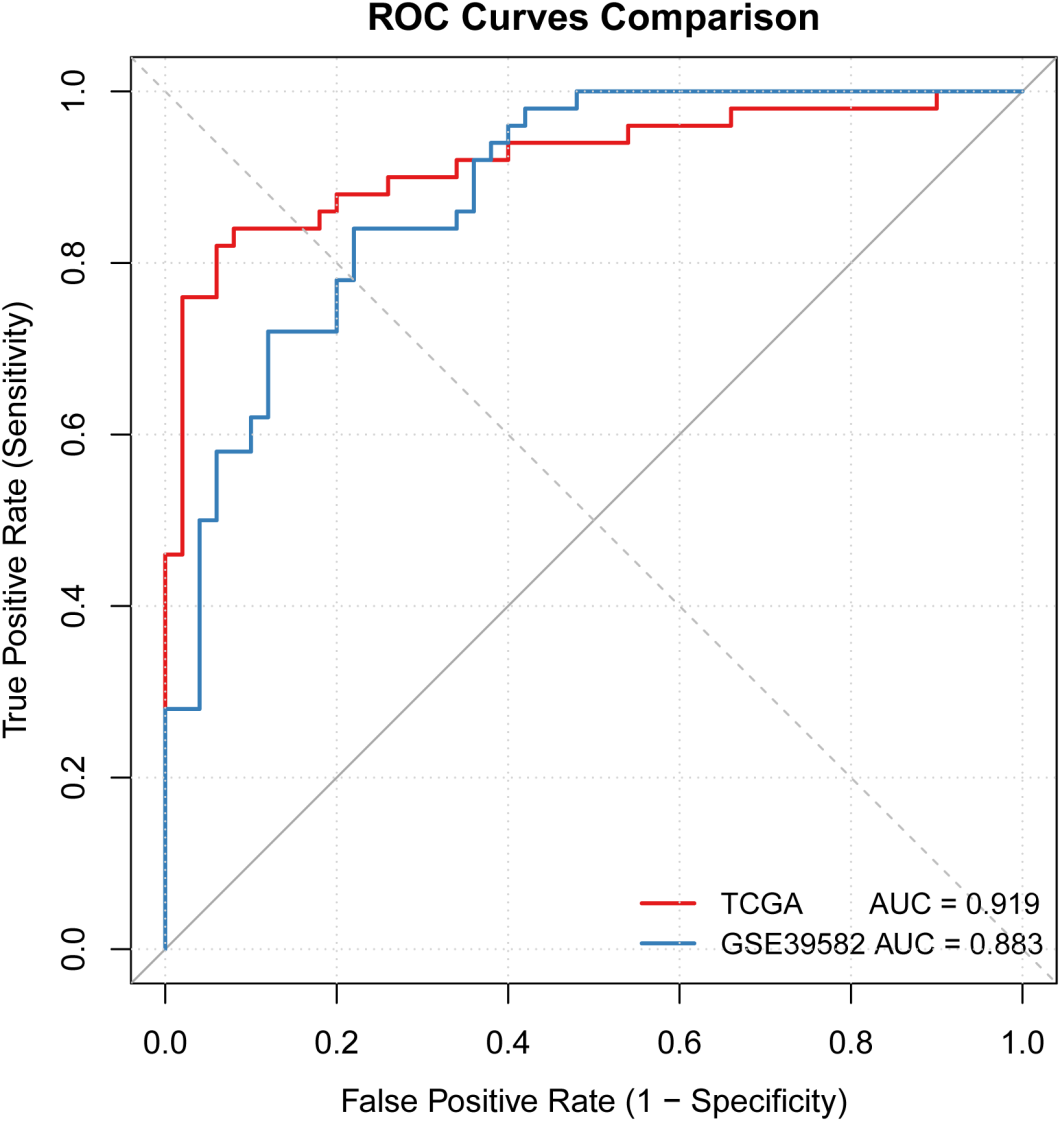
AUC analysis for the TCGA-CRC and the GSE39582 datasets.

Comparison of the diagnostic efficacy between the external datasets and the TCGA-CRC dataset showed that the AUC values of all datasets were above 0.81. The AUC values of the TCGA-CRC and GSE39582 datasets were 0.924 and 0.891, respectively, showing high consistency (**Figure 9**). This indicated that the diagnostic model had good generalization ability and could be applied to CRC samples from different regions and platforms.

### Diagnostic efficacy validation

**Figure 10:**
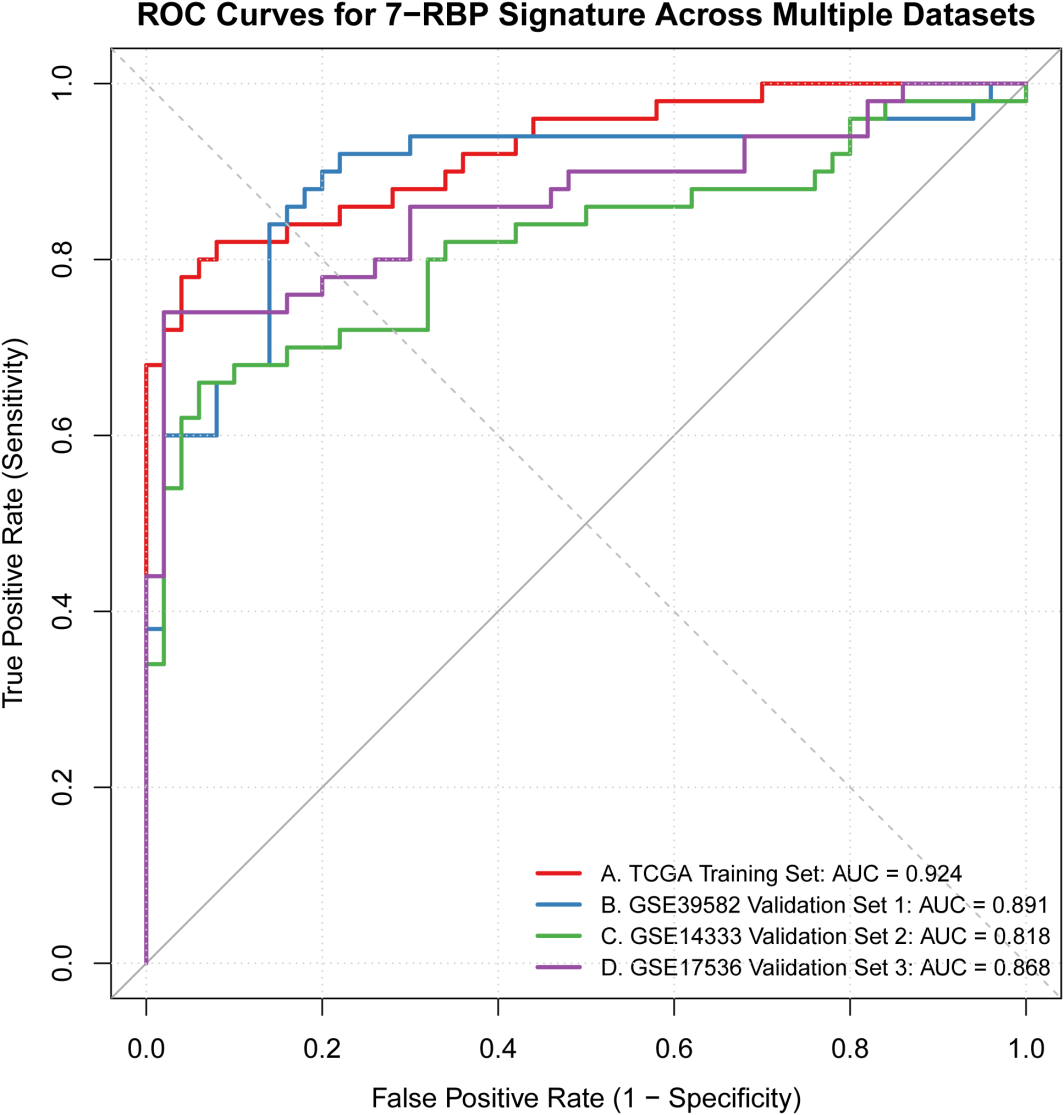
The diagnostic efficacy validation in TCGA-COAD, GSE39582, GSE14333, and GSE17536.

The diagnostic model of the seven hub RBP genes showed good diagnostic performance in four independent datasets (TCGA-COAD, GSE39582, GSE14333, GSE17536). The AUC values in the TCGA-CRC training set, GSE39582 validation set 1, GSE14333 validation set 2, and GSE17536 validation set 3 were 0.924, 0.891, 0.818, and 0.868, respectively. ROC curves showed that the model had stable diagnostic value in different cohorts with no obvious heterogeneity (**Figure 10**).

### Validation of the expression of hub RBPs by qRT-PCR and Western blot

**Figure 11:**
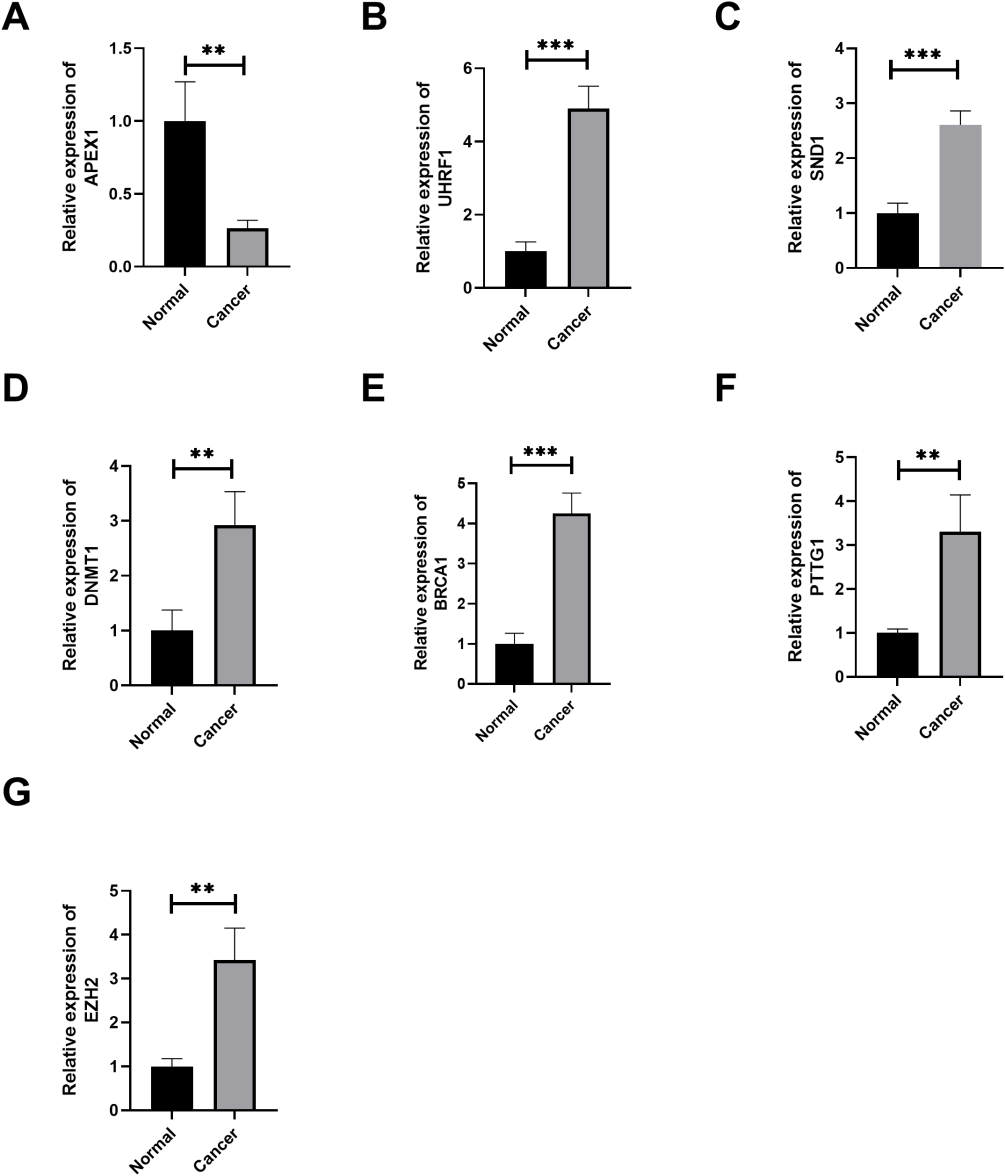
The verification of the hub RBPs in colorectal cancer tissues and normal tissues by qRT-PCR (*n* = 5). (**A–G**) Gene expression of APEX1, UHRF1, SND1, DNMT1, BRCA1, PTGG1, and EZH2 by qRT-PCR. **P < 0.01, ***P < 0.001.

**Figure 12:**
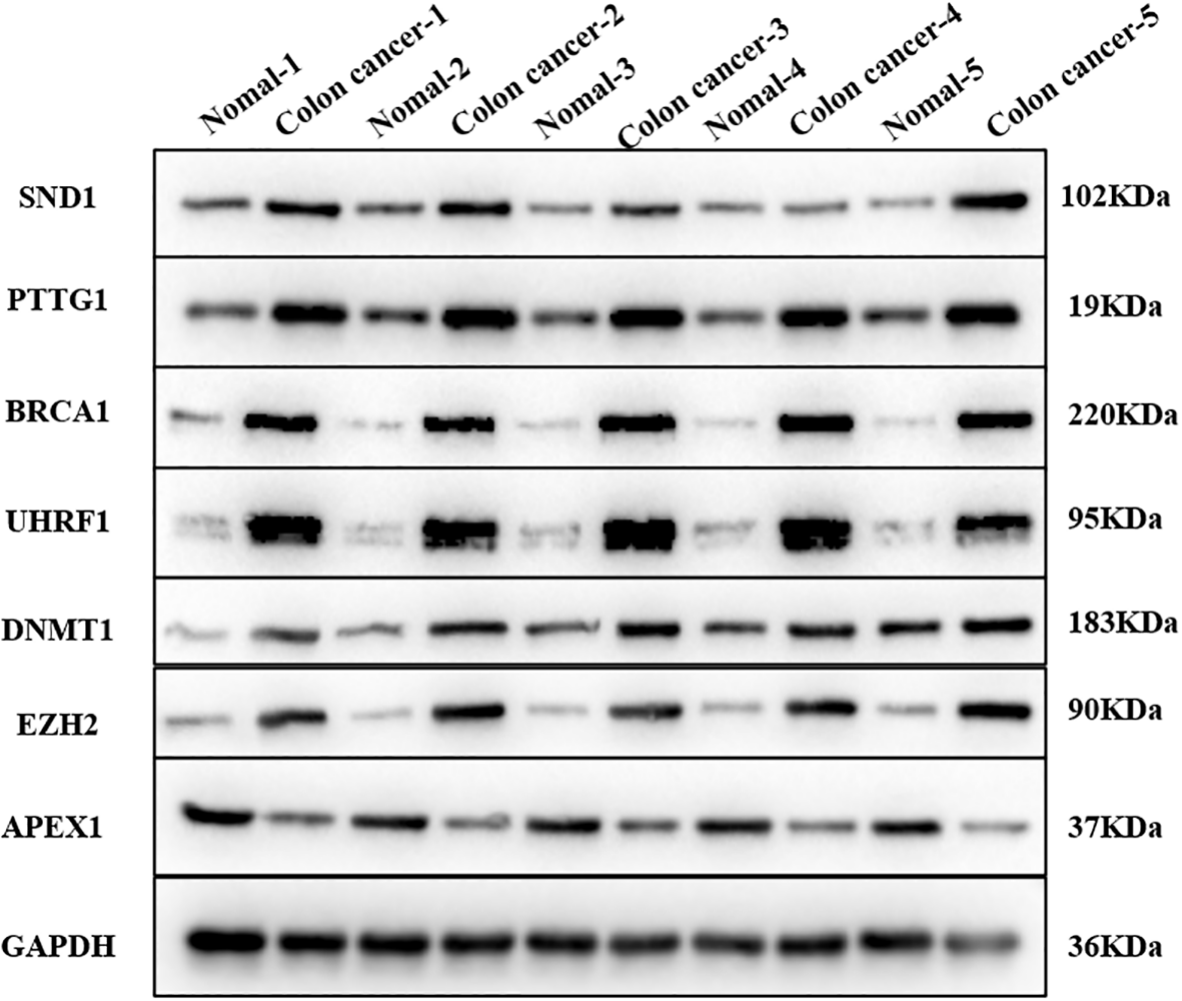
The verification of the hub IMRBPs in colorectal cancer tissues and normal tissues by WB (*n* = 5).

To enhance the credibility of our study, we chose to validate the expression of the hub RBPs. The results showed that the expression of the seven RBP genes was significantly associated with colorectal cancer tissues and normal tissues (**Figures 11**, **12**). We found that APEX1 was downregulated in colorectal cancer tissues (**Figure 11A**), while UHRF1, SND1, DNMT1, BRCA1, PTGG1, and EZH2 were upregulated in colorectal cancer tissues by qRT-PCR (**Figures 11B–G**). Furthermore, the WB results were consistent with the qRT-PCR results (**Figure 12**).

### Hub genes regulate ferroptosis mechanism in CRC

**Figure 13:**
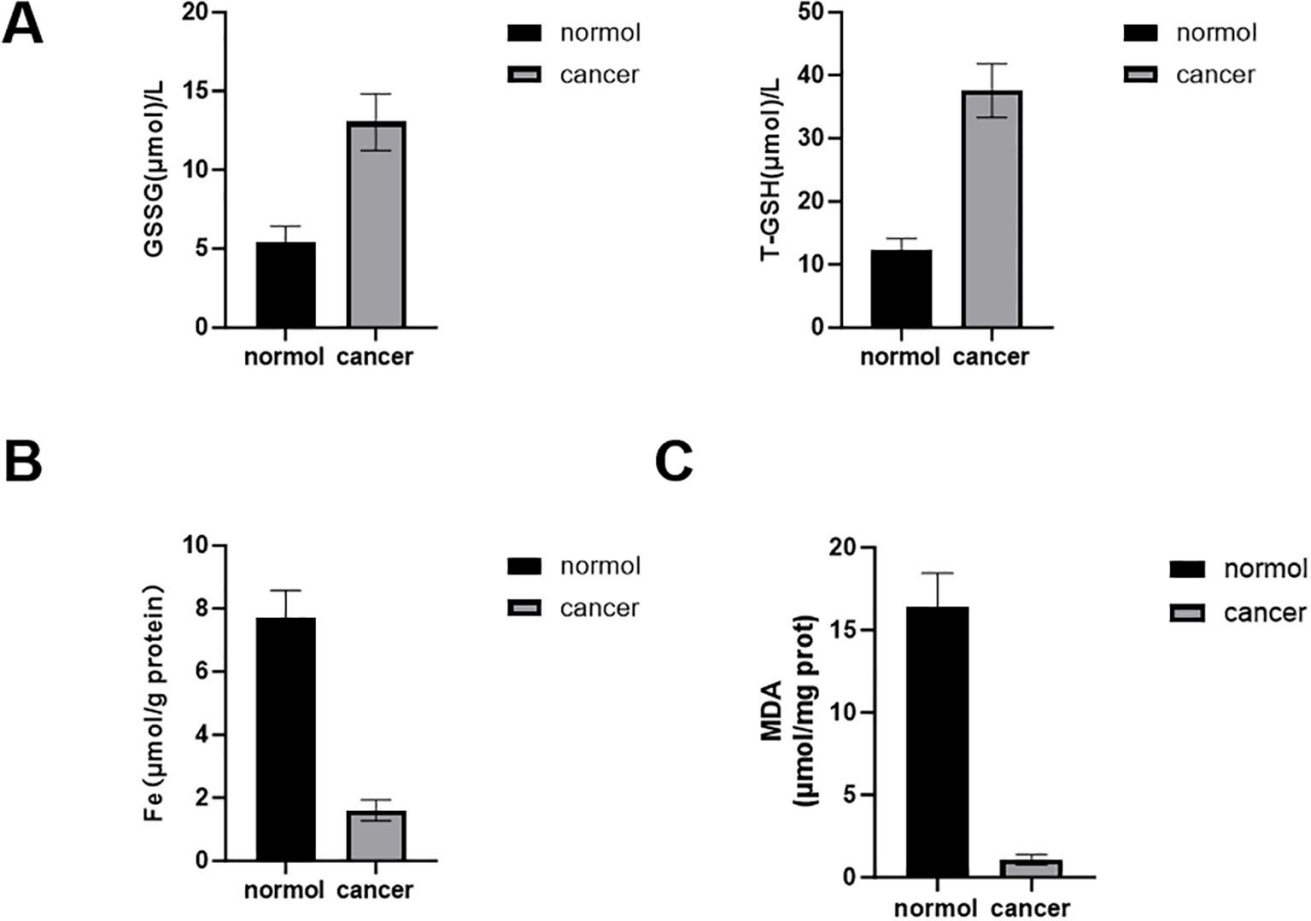
Effect of the expression of the seven hub IMRBPs on ferroptosis mechanism. **(A)** Glutathione assay. **(B)** Fe^2+^ level assay. **(C)** MDA assay.

To further investigate whether the hub genes were associated with ferroptosis mechanism in CRC, the GSH assay, Fe^2+^ level assay, and MDA assay were performed. We observed a higher GSSG/T-GSH level in CRC cancer tissues compared with the corresponding normal control (NC) groups by the GSH assay (**Figure 13A**). In addition, we determined the association between iron homeostasis and hub gene expression by measuring Fe^2+^ levels, and the result showed that the hub genes were negatively associated with the Fe^2+^ level in CRC tissues (**Figure 13B**). In the subsequent MDA assay, the relative MDA level was sharply reduced in CRC tissues compared to the NC group (**Figure 13C**). The above experiments showed that hub genes were associated with the multiple roles of suppressed ferroptosis, including the regulation of GSH/GSSG and Fe^2+^ levels. However, these correlative findings require functional validation to establish causal relationships.

## Discussion

Colorectal cancer is a malignant tumor that occurs in the colon and rectum. It usually originates in the lining cells of the colon or rectum and may spread to surrounding tissues and other organs. To date, there are a number of molecules and targeted immune drugs for the treatment and diagnosis of colorectal cancer (40); however, there is still a need for more molecules for the diagnosis and treatment of colorectal cancer. In this study, unique biomarkers associated with colorectal cancer were identified via bioinformatics analysis and machine learning, thus providing a novel perspective and potential therapeutic targets for colorectal cancer research.

Our findings should be contextualized within the broader landscape of CRC research. The potential protective role of GLP-1 receptor agonists against CRC, as suggested by epidemiological and preclinical studies (6), opens avenues for exploring their interaction with the molecular pathways identified in our work, particularly those involving ferroptosis and mitochondrial function. The significance of microRNAs in CRC is further emphasized by variants like miR-27 rs895819 and altered expression of miR-423, which correlate with cancer risk and progression (7). Our identified hub RBPs may interact with such miRNA networks, potentially forming regulatory axes that influence CRC phenotypes. Similarly, the involvement of the ribosomal protein L22-like 1 in CRC (8) underscores the importance of translational regulation in cancer, a process where RBPs are key players. The dysregulation of RPL22L1 might intersect with the functions of our hub RBPs, warranting future investigation. The network analysis approach we employed is strengthened by methodologies established in prior studies (9), which demonstrate the utility of integrated molecular networks in identifying critical regulators and biomarkers in complex diseases like cancer. Moreover, the linc01615/miR-491-5p axis represents a compelling example of how non-coding RNAs can modulate CRC metastasis and survival (10). It is plausible that the RBP and ferroptosis-related genes we identified participate in or are regulated by similar lncRNA–miRNA–mRNA–ceRNA networks, contributing to the intricate molecular tapestry of CRC.

This study obtained gene expression profile data from the TCGA-CRC database and the GSE39582 dataset. Subsequently, strict data preprocessing was carried out, which included the deletion of empty vector probes and the handling of probes corresponding to multiple genes, thereby ensuring the accuracy and reliability of the data.

The limma package was employed to screen for differentially expressed genes. Through a variety of analytical methods (such as WGCNA analysis), the construction of the miRNA-hub gene network and the TF-hub gene network, and the utilization of LASSO and SVM-RFE algorithms to screen for hub genes, the relationships between genes were explored in-depth from multiple aspects, enhancing the credibility of the research results.

The expression of the seven hub genes related to ferroptosis, mitochondria, and RBP identified in this study exhibited significant differences between colorectal cancer tissues and normal tissues. Among these genes, APEX1 was downregulated in colorectal cancer tissues, while the expression of BRCA1, DNMT1, EZH2, PTTG1, SND1, and UHRF1 was upregulated. These genes are likely to be involved in the occurrence and development of colorectal cancer and hold the potential to serve as biomarkers for the diagnosis and prognostic evaluation of colorectal cancer.

The expression patterns of the seven hub RBP genes (APEX1, BRCA1, DNMT1, EZH2, PTTG1, SND1, and UHRF1) in different datasets were visualized. Their abnormal expression in colorectal cancer implies that they may play a crucial role in the disease process. Further investigations into these genes will contribute to a more profound understanding of the pathogenesis of colorectal cancer.

Through the analysis of hub RBP gene subtypes, it was discovered that different subtypes differed in terms of stemness index (41), immune infiltration (42, 43), and other aspects. Additionally, 10 potential therapeutic drugs were screened out. This indicates that these biomarkers may be correlated with the clinical features of colorectal cancer, such as tumor stemness and immune microenvironment, providing a theoretical foundation for personalized treatment.

The study revealed that the GSSG/T-GSH level was higher in colorectal cancer tissues. Meanwhile, the hub genes negatively regulated the Fe^2+^ level in CRC tissues, and the relative MDA level was reduced. This suggests that the hub genes are associated with suppressed ferroptosis through various pathways, including the regulation of GSH/GSSG and Fe^2+^ levels, thereby potentially influencing the development of colorectal cancer. However, the current evidence is correlative, and future studies should include functional experiments such as gene knockdown/overexpression in CRC cell lines combined with ferroptosis inducers/inhibitors to establish causality. This finding presents a new potential target for the treatment of colorectal cancer, that is, treating colorectal cancer by intervening in the ferroptosis-related pathway.

### Limitations of the study

Although this study has achieved certain outcomes, it still has limitations. First, the association between hub genes and ferroptosis suppression remains correlative; functional validation through genetic manipulation in cell lines is needed to establish causality. Second, the PPI network analysis is descriptive and does not elucidate directed regulatory relationships; future studies should integrate CLIP-seq data to identify direct RNA targets of these RBPs. Third, while we validated the prognostic signature in external datasets, prospective validation in multicenter cohorts is warranted. The research samples mainly originated from specific databases and a limited number of clinical samples, which may introduce certain biases. Future studies should expand the sample size to cover patients of different ethnicities, regions, and clinical features, thereby improving the universality and representativeness of the research results.

## Conclusion

Through integrated bioinformatics and machine learning approaches, 14 ferroptosis-mitochondria-RBP-related genes (IMRBPs) were identified, among which seven hub genes show significant differential expression between CRC and normal tissues, which provides a new perspective and potential therapeutic targets for colorectal cancer research. However, further in-depth research is indispensable to promote its application in clinical diagnosis, treatment, and prognosis evaluation.

## Data Availability

The datasets presented in this study can be found in online repositories. The names of the repository/repositories and accession number(s) can be found in the article/supplementary material.

## References

[1] Eng C Yoshino T Ruíz-García E Mostafa N Cann CG O'Brian B . Colorectal cancer. Lancet. (2024) 404:294–310. doi: 10.1016/S0140-6736(24)00360-X, PMID: 38909621

[2] White MT Sears CL . The microbial landscape of colorectal cancer. Nat Rev Microbiol. (2024) 22:240–54. doi: 10.1038/s41579-023-00973-4, PMID: 37794172

[3] Biller LH Schrag D . Diagnosis and treatment of metastatic colorectal cancer: A review. JAMA. (2021) 325:669–85. doi: 10.1001/jama.2021.0106, PMID: 33591350

[4] Sinicrope FA . Increasing incidence of early-onset colorectal cancer. N Engl J Med. (2022) 386:1547–58. doi: 10.1056/NEJMra2200869, PMID: 35443109

[5] Zhang G Liu P Wang G He X Xu L Wang Y . Local tumor progression predictive model based on MRI for colorectal cancer liver metastases after radiofrequency ablation. Discov Med. (2024) 36:765–77. doi: 10.24976/Discov.Med.202436183.72, PMID: 38665025

[6] Tong G Peng T Chen Y Sha L Dai H Xiang Y . Effects of GLP-1 receptor agonists on biological behavior of colorectal cancer cells by regulating PI3K/AKT/mTOR signaling pathway. Front Pharmacol. (2022) 13:901559. doi: 10.3389/fphar.2022.901559, PMID: 36034798 PMC9399678

[7] Barahman M Alijanpour A Naseri A Miri S Firouzi F Shirinzadeh-Dastgiri A . Correlation between miR-27 rs895819 and miR-423 rs6505162 Polymorphisms and Susceptibility to Colorectal Cancer in the Iranian Population: A Case-Control Study. EJMO. (2024) 8(3):348–57. doi: 10.14744/ejmo.2024.56068

[8] Li C Du X Zhang H Liu S . Knockdown of ribosomal protein L22-like 1 arrests the cell cycle and promotes apoptosis in colorectal cancer. Cytojournal. (2024) 21:45. doi: 10.25259/Cytojournal_29_2024, PMID: 39737125 PMC11683392

[9] Li XY Xiang J Wu FX Li M . NetAUC: A network-based multi-biomarker identification method by AUC optimization. Methods. (2022) 198:56–64. doi: 10.1016/j.ymeth.2021.08.001, PMID: 34364986

[10] Xiao Y Hu F Li M Mo L Xu C Wang X . Interaction between linc01615 and miR-491-5p regulates the survival and metastasis of colorectal cancer cells. Transl Cancer Res. (2020) 9:2638–47. doi: 10.21037/tcr.2020.03.03, PMID: 35117623 PMC8798974

[11] ME LL . Understanding RNA-binding proteins. Semin Cancer Biol. (2022) 86:135–6. doi: 10.1016/j.semcancer.2022.06.015, PMID: 35787942

[12] Gerstberger S Hafner M Tuschl T . A census of human RNA-binding proteins. Nat Rev Genet. (2014) 15:829–45. doi: 10.1038/nrg3813, PMID: 25365966 PMC11148870

[13] Dreyfuss G Kim VN Kataoka N . Messenger-RNA-binding proteins and the messages they carry. Nat Rev Mol Cell Biol. (2002) 3:195–205. doi: 10.1038/nrm760, PMID: 11994740

[14] Mitchell SF Parker R . Principles and properties of eukaryotic mRNPs. Mol Cell. (2014) 54:547–58. doi: 10.1016/j.molcel.2014.04.033, PMID: 24856220

[15] Li W Deng X Chen J . RNA-binding proteins in regulating mRNA stability and translation: roles and mechanisms in cancer. Semin Cancer Biol. (2022) 86:664–77. doi: 10.1016/j.semcancer.2022.03.025, PMID: 35381329 PMC9526761

[16] Brownmiller T Caplen NJ . The HNRNPF/H RNA binding proteins and disease. Wiley Interdiscip Rev RNA. (2023) 14:e1788. doi: 10.1002/wrna.1788, PMID: 37042074 PMC10523889

[17] Zhao Y Mir C Garcia-Mayea Y Paciucci R Kondoh H LLeonart ME . RNA-binding proteins: Underestimated contributors in tumorigenesis. Semin Cancer Biol. (2022) 86:431–44. doi: 10.1016/j.semcancer.2022.01.010, PMID: 35124196

[18] Mehta M Raguraman R Ramesh R Munshi A . RNA binding proteins (RBPs) and their role in DNA damage and radiation response in cancer. Adv Drug Delivery Rev. (2022) 191:114569. doi: 10.1016/j.addr.2022.114569, PMID: 36252617 PMC10411638

[19] Jiang X Stockwell BR Conrad M . Ferroptosis: mechanisms, biology and role in disease. Nat Rev Mol Cell Biol. (2021) 22:266–82. doi: 10.1038/s41580-020-00324-8, PMID: 33495651 PMC8142022

[20] Zeng F Nijiati S Tang L Ye J Zhou Z Chen X . Ferroptosis detection: from approaches to applications. Angew Chem Int Ed Engl. (2023) 62:e202300379. doi: 10.1002/anie.202300379, PMID: 36828775

[21] Chen X Kang R Kroemer G Tang D . Broadening horizons: the role of ferroptosis in cancer. Nat Rev Clin Oncol. (2021) 18:280–96. doi: 10.1038/s41571-020-00462-0, PMID: 33514910

[22] Dixon SJ Olzmann JA . The cell biology of ferroptosis. Nat Rev Mol Cell Biol. (2024) 25:424–42. doi: 10.1038/s41580-024-00703-5, PMID: 38366038 PMC12187608

[23] Lei G Zhuang L Gan B . Targeting ferroptosis as a vulnerability in cancer. Nat Rev Cancer. (2022) 22:381–96. doi: 10.1038/s41568-022-00459-0, PMID: 35338310 PMC10243716

[24] Kim R Taylor D Vonderheide RH Gabrilovich DI . Ferroptosis of immune cells in the tumor microenvironment. Trends Pharmacol Sci. (2023) 44:542–52. doi: 10.1016/j.tips.2023.06.005, PMID: 37380530

[25] Nunnari J Suomalainen A . Mitochondria: in sickness and in health. Cell. (2012) 148:1145–59. doi: 10.1016/j.cell.2012.02.035, PMID: 22424226 PMC5381524

[26] Tabara LC Segawa M Prudent J . Molecular mechanisms of mitochondrial dynamics. Nat Rev Mol Cell Biol. (2024) 26(2):123–46. doi: 10.1038/s41580-024-00785-1, PMID: 39420231

[27] Suomalainen A Nunnari J . Mitochondria at the crossroads of health and disease. Cell. (2024) 187:2601–27. doi: 10.1016/j.cell.2024.04.037, PMID: 38788685

[28] Borcherding N Brestoff JR . The power and potential of mitochondria transfer. Nature. (2023) 623:283–91. doi: 10.1038/s41586-023-06537-z, PMID: 37938702 PMC11590279

[29] Chen V Yang M Cui W Kim JS Talwalkar A Ma J . Applying interpretable machine learning in computational biology-pitfalls, recommendations and opportunities for new developments. Nat Methods. (2024) 21:1454–61. doi: 10.1038/s41592-024-02359-7, PMID: 39122941 PMC11348280

[30] Addala V Newell F Pearson JV Redwood A Robinson BW Creaney J . Computational immunogenomic approaches to predict response to cancer immunotherapies. Nat Rev Clin Oncol. (2024) 21:28–46. doi: 10.1038/s41571-023-00830-6, PMID: 37907723

[31] Wang Z Tang W Yuan J Qiang B Han W Peng X . Integrated analysis of RNA-binding proteins in glioma. Cancers (Basel). (2020) 12(4):892. doi: 10.3390/cancers12040892, PMID: 32272554 PMC7226056

[32] Wilkerson MD Hayes DN . ConsensusClusterPlus: a class discovery tool with confidence assessments and item tracking. Bioinformatics. (2010) 26:1572–3. doi: 10.1093/bioinformatics/btq170, PMID: 20427518 PMC2881355

[33] Hanzelmann S Castelo R Guinney J . GSVA: gene set variation analysis for microarray and RNA-seq data. BMC Bioinf. (2013) 14:7. doi: 10.1186/1471-2105-14-7, PMID: 23323831 PMC3618321

[34] Yu G Wang LG Han Y He QY . clusterProfiler: an R package for comparing biological themes among gene clusters. OMICS. (2012) 16:284–7. doi: 10.1089/omi.2011.0118, PMID: 22455463 PMC3339379

[35] Charoentong P Finotello F Angelova M Mayer C Efremova M Rieder D . Pan-cancer immunogenomic analyses reveal genotype-immunophenotype relationships and predictors of response to checkpoint blockade. Cell Rep. (2017) 18:248–62. doi: 10.1016/j.celrep.2016.12.019, PMID: 28052254

[36] Zeng D Ye Z Shen R Yu G Wu J Xiong Y . IOBR: multi-omics immuno-oncology biological research to decode tumor microenvironment and signatures. Front Immunol. (2021) 12:687975. doi: 10.3389/fimmu.2021.687975, PMID: 34276676 PMC8283787

[37] Maeser D Gruener RF Huang RS . oncoPredict: an R package for predicting *in vivo* or cancer patient drug response and biomarkers from cell line screening data. Brief Bioinform. (2021) 22(6):bbab260. doi: 10.1093/bib/bbab260, PMID: 34260682 PMC8574972

[38] Malta TM Sokolov A Gentles AJ Burzykowski T Poisson L Weinstein JN . Machine learning identifies stemness features associated with oncogenic dedifferentiation. Cell. (2018) 173:338–354 e15. doi: 10.1016/j.cell.2018.03.034, PMID: 29625051 PMC5902191

[39] Wang H Wu Y Wang Z Chen Y Mo J Guan W . The LncRNA FEZF1-AS1 promotes tumor proliferation in colon cancer by regulating the mitochondrial protein PCK2. Oncol Res. (2021) 29:201–15. doi: 10.32604/or.2022.03553, PMID: 37304670 PMC10208053

[40] Di Nicolantonio F Vitiello PP Marsoni S Siena S Tabernero J Trusolino L . Precision oncology in metastatic colorectal cancer - from biology to medicine. Nat Rev Clin Oncol. (2021) 18:506–25. doi: 10.1038/s41571-021-00495-z, PMID: 33864051

[41] Hasan A Khan NA Uddin S Khan AQ Steinhoff M . Deregulated transcription factors in the emerging cancer hallmarks. Semin Cancer Biol. (2024) 98:31–50. doi: 10.1016/j.semcancer.2023.12.001, PMID: 38123029

[42] Hashimoto S Kishimoto T . Roles of RNA-binding proteins in immune diseases and cancer. Semin Cancer Biol. (2022) 86:310–24. doi: 10.1016/j.semcancer.2022.03.017, PMID: 35351611

[43] Turner M Diaz-Munoz MD . RNA-binding proteins control gene expression and cell fate in the immune system. Nat Immunol. (2018) 19:120–9. doi: 10.1038/s41590-017-0028-4, PMID: 29348497

